# American Medical Association

**Published:** 1858-09

**Authors:** 


From the North American Medico Chirurgical Review.

### *American Medical Association.*

The recent Meeting of the American Medical Association at Washington proved, as was anticipated, to be one of the most agreeable, as it was also one of the largest, assemblies of the kind that has ever been convened. The number of delegates present was nearly or quite five hundred, representing, with only two or three exceptions, every State in the Union. Even California had her member in the person of Dr. Harvey, who was one of the few passengers saved, and the only one of five physicians on the occasion of the sinking of the ill-fated steamer Central America, on her way from the Isthmus to New York, a few months ago. The proceedings of the meeting, although possessing a certain kind of interest to those who are fond of medical politics, we do not think are of sufficient importance to justify the space which their publication in our pages would require. The number and value of contributions, as well as can be ascertained, will equal, if not exceed, those of any previous session ; and when the volume of transactions shall appear, we shall take pleasure in laying before our readers abstracts of the most interesting papers.

The meeting was held in the lecture-room of the Smithsonian Institute, where the delegates were welcomed to Washington in a pleasant speech by Dr. Harvey Lindsay, Chairman of the Committee of Arrangements. The retiring President, Dr. Paul F. Eve, then delivered an address, consisting mostly of a sketch of the history of the Association, and a statement of what it had accomplished, closing with a few decided, but we think rather problematical, assertions concerning its moral power. Upon this last theme, and in allusion to the controlling influence assumed for the Association over medical education, and particularly over the medical colleges of the country, the worthy President made use of the following extravagant language :

"It has," he says, "but to speak on this point, and it will be obeyed ; for it is now too late for any physician to oppose, or any medical college to set at defiance, the moral power of this body." Now, if we mistake not, this all-powerful body has repeatedly lifted its trumpet-tongued voice against the medical schools,—its messengers, in the form of committee-men, raising themselves as high as the two short stirrups of the saddle on which they sit will permit, and in true Quixotic tones summoning the windmills of the plain to lay down their arms and surrender ; but the latter, not excepting the one in which the President himself is interested, unimpressible by what seems to be only a *vox et prœterea nihil*, continue to turn their sails to the favoring breeze, from whatever quarter it may come, and go on grinding out doctors in the old tic tac style, stopping only now and then to have a rotten or worn out cog replaced with one of better stuff, or to have the stones set a little closer, to suit the increasing demand for a finer product.

We have no idea that the Association, as at present organized, consisting as every body knows, of a large majority of what are called, curiously enough, *lay* members of the profession, and evincing as it does, in a greater or less degree, a sentiment of hostility toward the medical colleges, will ever succeed in accomplishing its high and worthy object in the matter of medical education, until it shall assume a more deferential tone toward those in whom the power practically resides. It is the purest fustian gravely to announce to the medical faculties throughout the country that they dare not disobey the commands of the National Association; when it is a well known fact that the latter, notwithstanding the great moral power claimed foi it by the late President, has not succeeded, after a labor of ten long years, in incorporating a single one of its propositions among the requirements for graduation heretofore agreed upon by the leading schools. But we think the Association is coming to its senses upon this question, for, at the meeting just held, a committee resolution was adopted, requesting all the schools to send their delegates next year to a special meeting, to be held at the place agreed upon by the Association, one day in advance of the latter, to concert such matters as they may deem best for the improvement of the present system of public teaching; the result of their deliberations to be presented in a report to the Association. In other words, the Association has appointed a committee, consisting of all the delegates of the medical schools, to make a report upon the subject of medical education at the next meeting. If the schools, and more especially those that are generally looked upon as the leading institutions of the kind, will take this matter in hand, some good may posibly be accomplished; and yet it requires no prophetic vision to discern almost insurmountable difficulties in the way. We refer to only a single one, namely, the question of clinical instruction. Bedside teaching is admitted, by all sober-thinking minds, to be essential to an adequate course of instruction, before the student applies for graduation, and yet not one-half the schools are enabled to offer anything more in this way than the occasional introduction of a patient into the lecture-room of the college. But still, we hope for something, undefined though that something be to our minds at present, and shall lend our feeble efforts in promoting the success of the experiment.

The first and by far the most exciting topic discussed at the recent meeting, was the offence of Dr. Reese, of New York. and Dr. Bryan, of Philadelphia, against the code of medical ethics,—these two gentlemen having given letters to the notorious Dr. McClintock, recommending him for the place of Physician-in-Chief to the Philadelphia Hospital. Dr. Reese took the initiative by appologizing to the Association, of which, it will be remembered he was one of the Vice Presidents, which apology was, upon motion of one of the Philadelphia delegation, immediately accepted; but being deemed by many insufficient it was subsequently reconsidered, and after much warm discussion and the exhibition of no little personal feeling upon the part of several members, Dr. Reese consented to the following as a substitute :—"The undersigned regrets that he certified to the professional qualifications, for Blockley hospital, Philadelphia, of an expelled member of this body, and hereby offers this apology for his departure from the ethical code."

This was considered as satisfactory by all, and Dr. Bryan having walked over the path that had been thus made by Dr. Reese, the usual business of the meeting was proceeded with.

Previously to the final settlement of the ethical case, however, the committee on nomination made their report, which was accepted, and the following named gentlemen were unanimously elected to serve for the unsuing year :—*President* Dr. Harvey Lindsley, of Washington. *Vice Presidents*, Drs. G. L. Sutton, of Kentucky; Thomas C. Edwards, of Iowa; Josiah Crosby, of New Hampshire; W. C. Warren, of North Carolina. *Secretaries*, Drs. A. J. Semmes, of Washington; and S. M. Bemiss, of Louisville. *Treasurer*, Dr. Caspar Wister, of Philadelphia. The next place of meeting fixed upon was Louisville, Kentucky.

The Committee on Prize Essays reported that they had received but three essays, of which two were approved as worthy of prizes. The first, entitled "On the Clinical Study of the Heart's Sounds, in Health and Disease," was found upon opening the sealed packet, to have been written by Dr. Austin Flint, Sen., of Buffalo. The second "On Vision, and some of its Anomalies, as Revealed by the Ophthalmoscope," was in like manner, found to have been contributed by Dr. Montrose A. Pallen, of St. Louis. This is the second time that Dr. Flint has received the first prize, and the third time that Buffalo has had the honor of producing the successful candidate,—Dr. Hamilton having once carried away the first prize.

There was no report from the committee on Medical Education, of which Dr. Norris, of Philadelphia, was Chairman.

Dr. Palmer, of Michigan, made a report on Medical Literature, which will claim our attention hereafter. From the special committee numerous reports were received; among which, besides several upon Epidemics, may be mentioned one from the pen of Dr. Bemiss, of Louisville, "On the Influence of Marriages of Consanguinity upon Offspring; one on "Spontaneous Umbilical Hemorrhage of Newly-born Infants," by Dr. Foster Jenkins; one by Dr. Stephenson, of New York, on "Cataract, etc.;" and one by Dr. Campbell of Augusta, on "Nervous concomitants of Febrile Diseases." Several voluntary papers were also presented, and referred to a special committee, with authority to send them to the Committee of Publication.

The amendments to the constitution, proposed at the last meeting were all rejected. We believe, however, that the Association will, one day or other, see the propriety of one of the modifications thus voted down, and will consent to its adoption. We refer to the proposition to amend so as to allow of the appointment of one permanent Secretary, whose duty it shall be to attend all the meetings, and preserve the archives of the Association.

The profession and citizens of Washington entertained the delegates with princely munificence and the most generous-hearted hospitality. Besides a special reception at the White House, and one by the Hon. Mr. Douglas, and evening parties by Drs. May, Tyler, Riley, Miller, Johson, and Garnett, an excursion was provided to Mount Vernon, the day after the adjournment, and this was followed by a magnificent *planked-shad dinner* on the banks of the Potomac within sight of the house of Washington.

We regret that the Association again stultified itself in selecting the presiding officer from among the physicians of the place of meeting. We are quite sure that a large number of the thinking and more influential members are opposed to the practice, and would have rejoiced if a new era, in this respect, had been inaugurated at Washington, of all places the most fitting for such a change.

Another circumstance struck us with regret, and that is, that the election of officers is not postponed until near the close of the session. If this plan were adopted, the president would always have ample time to acquaint himself with the manner of presiding over such an assembly; and in this way no one can doubt that business of the Association would be conducted with much greater dignity and despatch. A proposition to amend the constitution so as to admit of this change was offered by the senior editor of this journal at St. Louis, in 1854, but was clamorously voted down at the ensuing meeting.

